# Amorphous multimetal based catalyst for oxygen evolution reaction

**DOI:** 10.1007/s43939-024-00087-5

**Published:** 2024-06-25

**Authors:** Zishuai Zhang, Daniela Vieira, Jake E. Barralet, Geraldine Merle

**Affiliations:** 1 Faculty of Medicine, McGill University, Montreal, Qc H3A 0C5 Canada; 2 Chemical Engineering Department, Polytechnique Montréal, Montreal, QC H3C 3A7 Canada

## Abstract

The development of efficient, low-cost water splitting electrocatalysts is needed to store energy by generating sustainable hydrogen from low power clean but intermittent energy sources such as solar and wind. Here, we report a highly sustained low overpotential for oxygen evolution reached by the unique combination of three metals (NiCoV) prepared from a simple low temperature auto-combustion process. The amorphous multimetal oxygen evolving catalyst could be stably coated on a stainless-steel support using a tribochemical particle blasting method to create an oxygen evolution reaction (OER) electrode with a low overpotential of 230 mV at 10 mA cm^−2^ and a low Tafel slope of 40 mV dec^−1^. In addition to their low overpotential, this oxygen evolving electrocatalyst preserved performance demonstrating a stability after 10 h at the technologically relevant current density and without any surface morphology alteration. Given the importance of sustainable hydrogen production, the development of this new OER catalyst points the way to removing a key technical bottleneck for the water splitting reaction and could offer a route to cost reduction and lowering hurdles to more widespread adaptation of electrolyser technologies for hydrogen production.

**Supplementary Information:**

The online version contains supplementary material available at 10.1007/s43939-024-00087-5.

## Introduction

Water electrolysis is an attractive option to store electrical energy from sustainable renewable energy sources [1]. Water splitting involves two simultaneous electrochemical reactions; the cathodic reduction of protons into hydrogen and an oxidation reaction at the anode generating oxygen gas [2]. Being a four-electron oxidative reaction, the oxygen evolution reaction (OER) is a more sluggish reaction compared to its cathodic counterpart, the hydrogen evolution reaction (HER) [3]. To improve the overall efficiency of the electrolysis process, both RuO_2_ and IrO_2_ have been considered as benchmark OER electrocatalysts due to their high electrocatalytic activities towards OER [4]. Unfortunately, the high cost of Ir and Ru (Ru = $575 per Troy Ounce and Ir = $4,150 per Troy Ounce; data from BASF Metal Prices Jan 2022) hinders their competitiveness and is a major barrier to their widespread implementation [5, 6]. Consequently, research has focused on the development of nonprecious metal electrocatalysts with similar performance. Previous studies demonstrated that the state-of-the-art 3d‐transition‐metal (TM) based catalysts exhibited comparable OER activities to RuO_2_ and IrO_2_ in the alkaline media [7, 8]. In this regard, electrocatalysts based on TM elements are of great practical promise for industrial-scale applications. However, complex surface engineering and fine-tuned morphology designs are often required for high activities [9–11], which made it challenging to maintain the low cost (despite the use of earth-abundant elements) and high catalytic performance when it comes to the mass production. We previously developed a low temperature auto-combustion process to prepare a stable and efficient amorphous mixed metal oxide catalyst based on cobalt and vanadium [12]. Although this bimetallic catalyst showed high OER activity (an overpotential of 300 mV at 10 mA cm^−2^; Tafel slope of 66 mV dec^−1^), the high cost of Co ($70,000/ton, Trading Economics, Jan 2022) limits its application. Replacing Co with relatively cheaper Ni (($22,000/ton, Trading Economics, Jan 2022) could be a potential strategy to lower the overall cost. Furthermore, the atomic-level introduction of heterogeneous atoms affects the electronic structure – for example—Ni sites have been proven to be effective to facilitate electron transfer through the promotion of the bridge of Ni–O-M (M is the second metal site such as Fe) that create spin channels [13]. A notable feature of this described process was it enables the systematic tuning of the composition with different dopant (i.e., Ni) concentrations. In this work, we systematically studied ternary catalysts (consisting of Ni, Co and V) produced from the low-cost auto-combustion process and achieved superior OER activity (230 mV overpotential at 10 mA cm^−2^ and Tafel slope of 40 mV dec^−1^). Attachment of gas evolving catalysts to conductive substrates is not trivial as bubble formation can cause spalling [15]. The most common anode electrode in an alkaline electrolyzer is metal mesh (e.g., nickel, steel meshes) based electrodes [16]. The effective performance of a catalyst strongly depends on the surface structure, the performance is therefore diminished when incorporating polymeric binders that may block the pores and/or insulate the electrocatalytic site to construct the final electrode [17]. Moreover, the stability of binder-contained electrodes (fabricated via spray coating, drop casting, screen printing etc.) is still arguably poor due to the loss of catalyst during operation. Here, we developed a tribochemical coating technique achieving the strong adhesion through localized energy transferred between a high-speed catalyst coated particle and metal substrates [18]. The trimetallic coating deposited on stainless-steel mesh demonstrated high efficiency (40 mV dec^−1^) for the OER activity in the alkaline condition with a surprisingly low overpotential (230 mV at 10 mA cm^−2^) comparing favorably with RuO_2_ (73 mV dec^−1^; 190 mV at 10 mA cm^−2^) and high stability (no loss of OER performance after 10 h chronoamperometric measurements).

## Experimental

### Preparation of catalysts

The electrocatalysts were prepared through citrate–nitrate auto-combustion (CNA) method [12]. Cobalt (II) nitrate hexahydrate (Sigma Aldrich, > 99%), nickel (II) nitrate hexahydrate (Sigma Aldrich, > 99%) and vanadium (IV) oxide sulfate hydrate (Sigma Aldrich, 97%) were dissolved successively in milliQ water. After complete dissolution, citric acid was added to the mixture, and then the prepared solution was heated on a hotplate at ~ 80 °C overnight to remove the excessive water, resulting in brownish foam. Finally, the produced foam was further dried at 120 °C for 20 h followed by calcination at 250 °C for 3 h. Various compositions of catalysts were synthesized by using the same protocol.

The dried fine powders (**NiCoV**) were mixed with Al_2_O_3_ in a 1:20 ratio (w/w) using a mortar and pestle until uniform in color and then added to a sandblasting nozzle. Stainless steel meshes were coated by manually rastering the nozzle across the desired area until no bare steel was visible. In addition to bare stainless steel meshes and **NiCoV** coated meshes, commercial RuO_2_ nanoparticle (anhydrous, fisher scientific) coated meshes were fabricated using the same protocol.

### Characterization

Scanning electron microscopy (SEM) (Crossbeam 340, Zeiss, Oberkochen, Germany) with energy dispersive X-ray spectroscopy (XMaxN, Oxford-Instruments, Wiesbaden, Germany) and transmission electron microscopy (TEM, Tecnai F20) with an electron accelerating voltage of 200 kV were used to characterize the morphology of the catalyst support microparticles and meshes. The surface elemental composition of NiCoV and atomic bonding information were detected with XPS (Thermo Scientific K-Alpha). X-ray diffraction (XRD) analysis was carried out on a Bruker D8 Discovery instrument operating at 40 kV and 20 mA.

All electrochemical tests were performed using a potentiostat (VersaStat 4, Princeton Applied Research) and a three-electrode electrochemical cell. For testing the meshes, a saturated calomel electrode (SCE), platinum wire, and a stainless-steel mesh were used as the reference, counter and working electrodes, respectively. Rotating disk working electrodes (diameter of 4.9 mm) were used for *ex-situ* testing. The meshes were rinsed three times with milliQ water before starting the electrochemical testing. EIS was performed with potential perturbation of 10 mV within a frequency range of 10^5^–10^−1^ Hz, spaced logarithmically (120 per 10 decades).

## Results and discussion

**Fig. 1:**
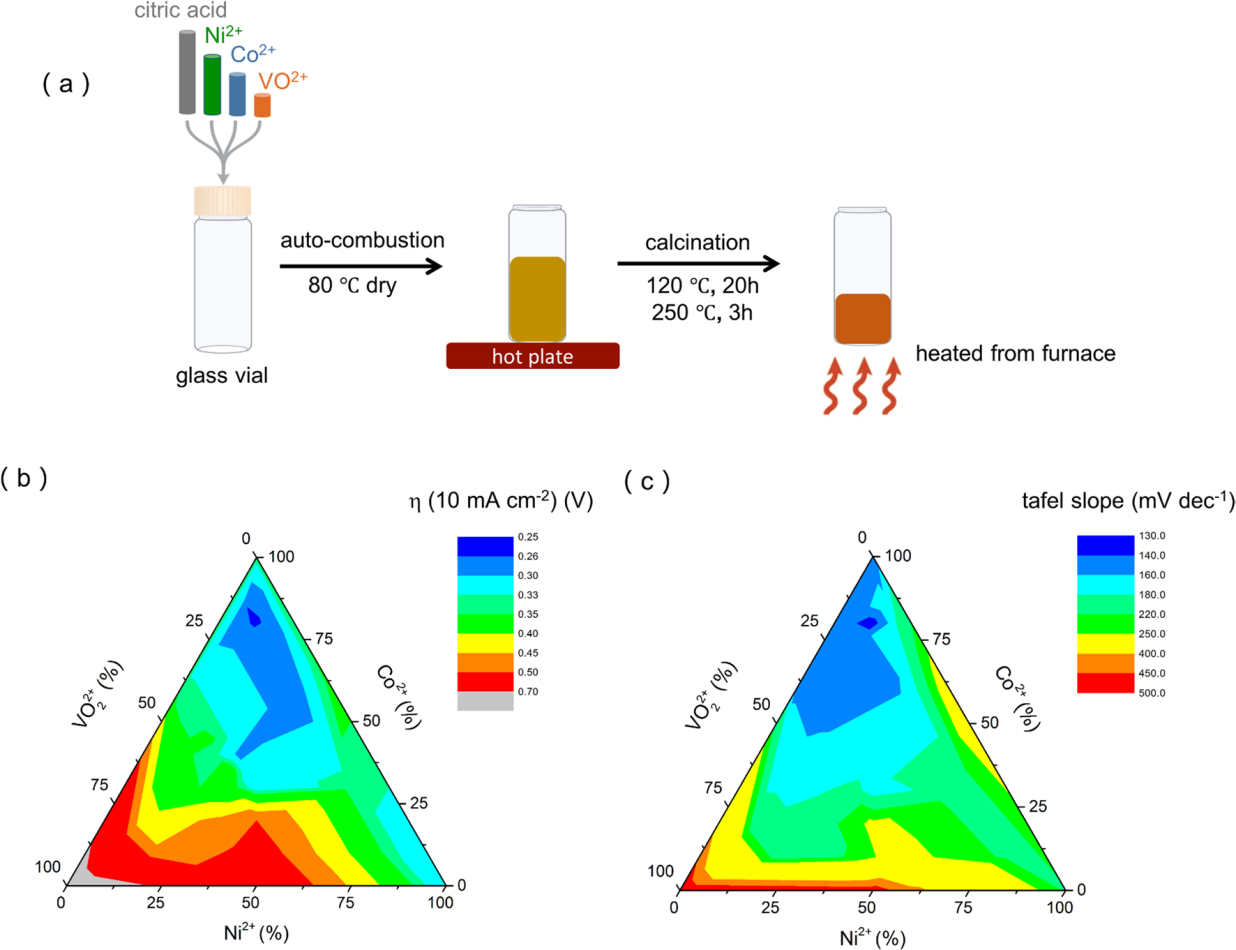
Preparation and performance of the trimetallic catalysts. **a** schematic showing steps in preparation of NiCoV catalyst; **b** ternary diagram for exploring the relationship between different composition of Ni-Co-V (with different atomic percentage) and their corresponding ŋ (10 mA cm^−2^) and **c** tafel slope values; the best performing composition is 10 at.%: 80 at.%: 10 at.% (Ni^2+^: Co^2+^: VO^2+^). Thirty-four samples were tested and the experiments were carried out in 1 M KOH without *iR* correction (see details in Table S1). As comparison, single metal oxide samples (defined as **Ni**, **Co** and **V**) and binary metal oxide samples (defined as **NiCo**, **CoV** and **NiV**) were synthesized as control samples through the same protocol (Table S2, details in Supporting Information)

We screened the electrocatalytic performances on a glassy carbon electrode in 1 M KOH electrolyte in a three-electrode system. Two activity metrics were used as the benchmarks in this study: overpotential value at 10 mA cm^−2^ (defined as ŋ (10 mA cm^−2^)) and Tafel slope value [19]. A lower overpotential value leads to a higher energy efficiency, and a lower Tafel slope value indicates that a same current density is reached at a lower potential because of a higher charge transfer coefficient [20]. The ternary diagrams were plotted and the results indicated the best OER activities were obtained for 1) a high Co^2+^ concentration (up to 80 at.% max.) and 2) VO^2+^ concentration range from 10 at.% to 40 at.% (Fig. 1b, c). The lowest overpotential of 250 mV was achieved at the atomic ratio of 10%: 80%: 10% (Ni^2+^: Co^2+^: VO^2+^) (defined as **NiCoV**), which also showed the lowest Tafel slope of 130 mV dec^−1^ (Fig. 1b, c). This performance metrics are comparable to the commercial RuO_2_ nanoparticles (ŋ = 230 mV at 10 mA cm^−2^; 132 mV dec^−1^) (Figure S1).

The ternary metallic electrocatalysts were prepared at low temperature via a citrate–nitrate auto-combustion process (Fig. 1a), in which citric acid (CA) acts as fuel and complexing agent and metal nitrates as the metal ion and oxidant sources [12]. The highly porous foam residue was formed after the combustion at 80 °C due to the vast amount of gas formation (e.g., NO_x_, CO_2_, CO etc.). The product was further calcined at higher temperatures (250 °C) to remove water and unreacted CA in a furnace. Thirty-four electrocatalyst compositions were synthesized, with different Ni^2+^: Co^2+^: VO^2+^ ratios to identifythe most electroactive electrocatalyst (Fig. 1b, c, details in Table S1).

**Fig. 2:**
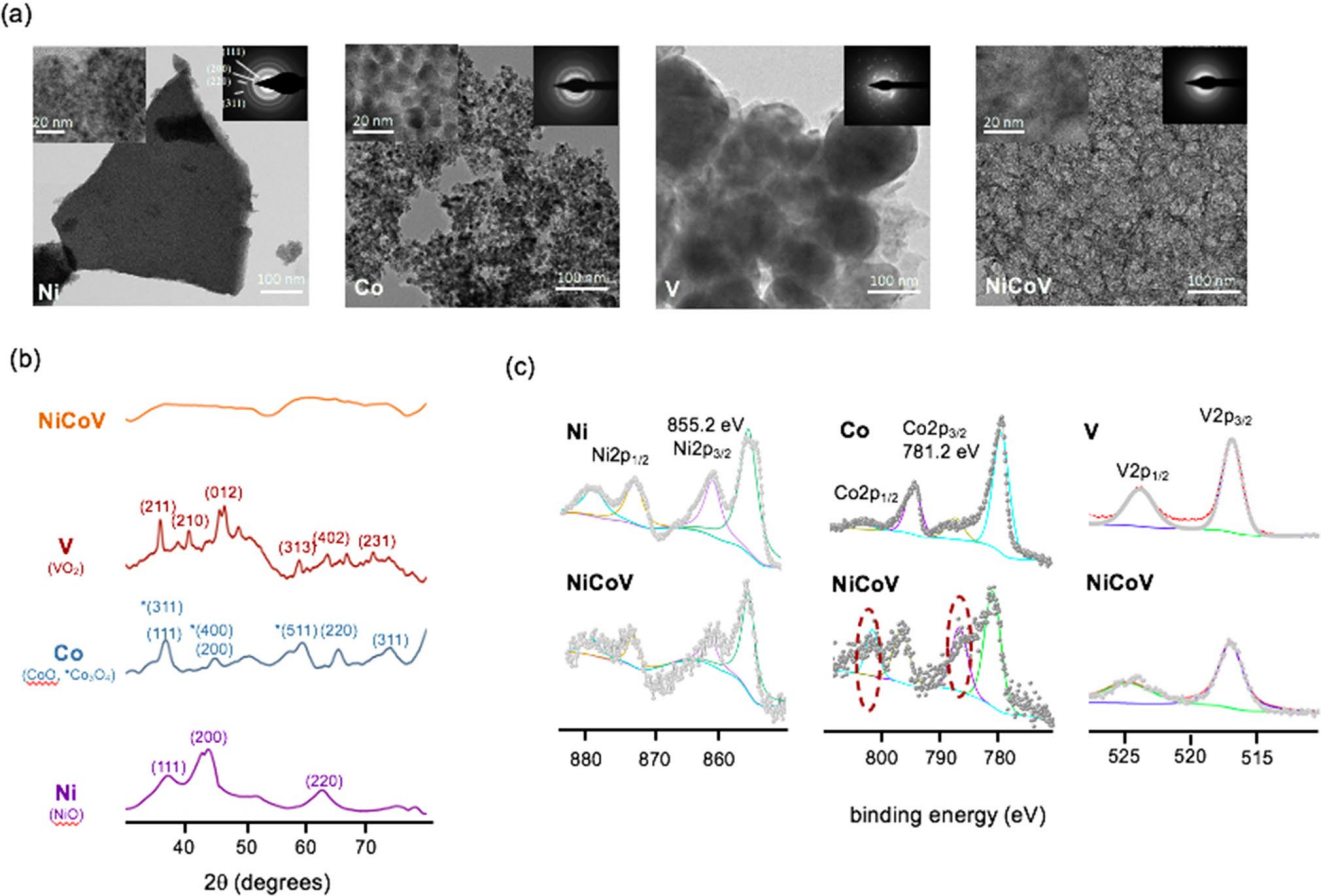
Characterization of the **NiCoV** and the control (**Ni**, **Co**, **V**) catalysts. **a** TEM images (inset high-resolution TEM images and SAED patterns) and **b** XRD patterns of the **Ni**, **Co**, **V** and **NiCoV** catalysts, respectively; **c** high resolution XPS spectra of Ni 2p for the **Ni** and **NiCoV** catalysts, Co 2p for the **Co** and **NiCoV** catalysts (the red dotted ovals indicate the signature satellite signals for Co^2+^), and V 2p for the **V** and **NiCoV** catalysts

Scanning and transmission electron microscopy (SEM and TEM) were employed for morphology and microstructure analysis. The **NiCoV** sample appeared to be large platelets with a smooth surface and a distinguished glassy layer structure (Fig. 2a, S2). Selected area electron diffraction (SAED) patterns revealed that the **NiCoV** and **NiV** electrocatalysts were mostly made of amorphous layers (Figs. 2a, S3), whereas the rest control catalysts (**Ni**, **Co**, **V**, **NiCo** and **CoV**) were composed by highly crystallized particles (Fig. 2a, S3). We assume vanadyl ion can stabilize the Ni-based metal oxides in its amorphous form and prevent it from crystallization, which is in agreement with the previous study [14]. It must be noted that Ni or Co ions cannot be crystalline inhibitors since NiCo is highly crystallized (Figure S3). X-ray diffraction (XRD) analysis confirmed that the crystal structures of **Ni**, **Co** and **V** match NiO, CoO and Co_3_O_4_, VO_2_ patterns respectively (Fig. 2b). As indicated by the TEM investigation, the presence of vanadyl ion (**NiV** and **NiCoV**), decreased drastically the crystallinity of the samples (Figs. 2b, S4). This phenomenon can be attributed to the vanadyl ions distorting the adjacent coordination of Ni, Co with O atoms, inducing lattice mismatch and the overall amorphous structure [14].

**Fig. 3:**
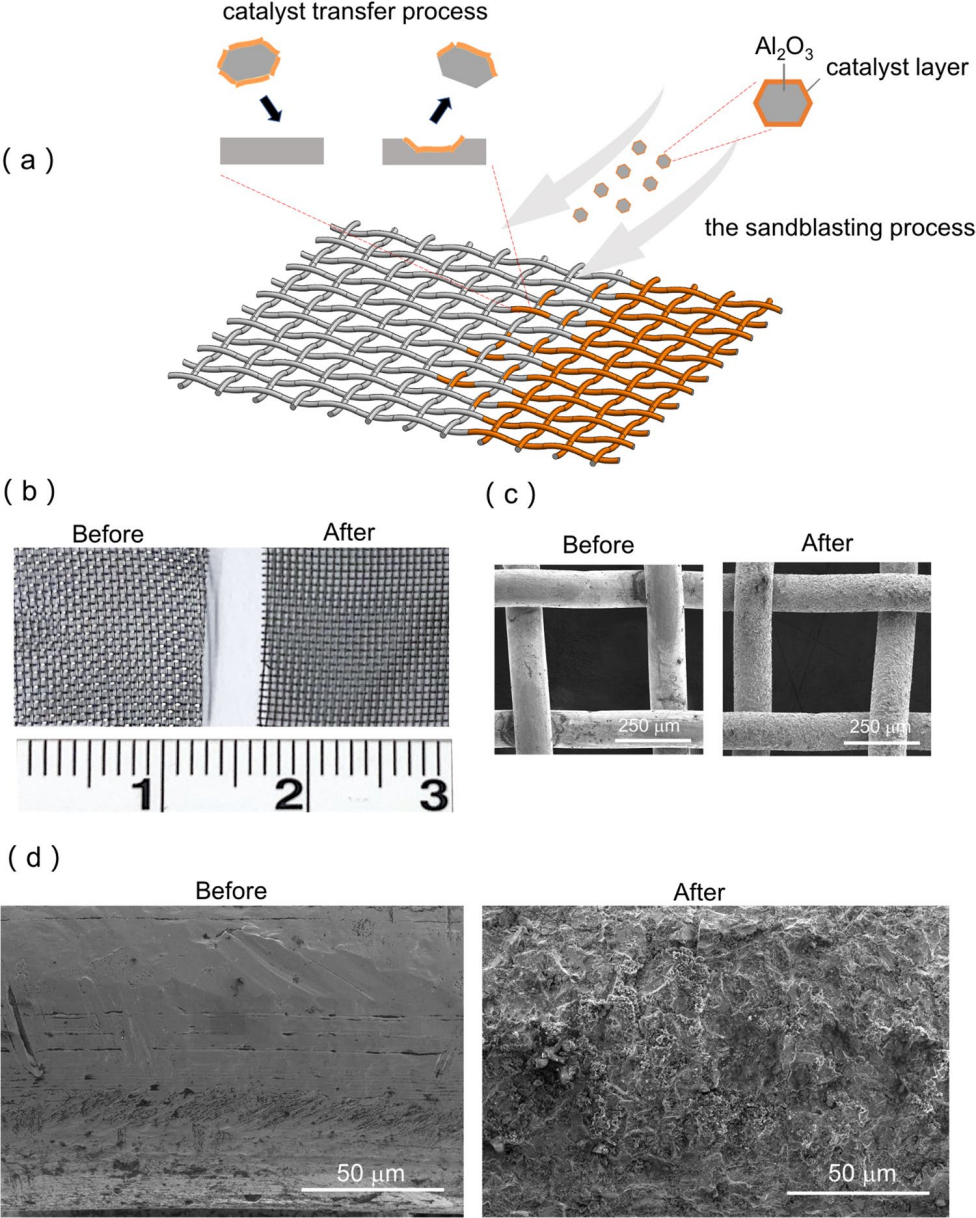
Formation of tribochemical coatings. **a** schematic of the transfer of catalyst skin from corundum to stainless steel substrate during sandblasting; **b** photograph of stainless steel meshes and **c** the SEM images of the meshes before and after sandblasting; **d** higher magnification SEM images of meshes before and after sandblasting

X-ray photoelectron spectroscopy (XPS) was carried out to determine the correlation between the OER activity and the oxidation states (Fig. 2c). The high-resolution spectra of Co 2p, Ni 2p and V 2p were collected for **NiCoV** and the control group (**Co**, **Ni** and **V**). Co_3_O_4_ was found to be the main species formed for the **Co** (Co 2p_3/2_ 779.7 eV; O 1 s 529.4 eV), while the two strong signature satellite signals at 787 eV and 803 eV indicated the presence of CoO in the **NiCoV** catalyst (Figs. 2, S5) [23]. A previous study has shown that the Co^2+^ is more catalytic active towards water oxidation due to the formation of cobalt oxyhydroxide (CoOOH) during catalysis [24]. Notwithstanding, we did not observe distinct Ni 2p and V 2p spectra between the **NiCoV** and the corresponding control samples: NiO in + 2 valence states (indicated by 17.50 eV energy difference between Ni 2p_1/2_ and Ni 2p_3/2_) [22] and VO_2_ in the + 4 valence state were determined in both NiCoV and the control groups (i.e., **Ni** and **V**).Traditionally, polymeric binders (e.g., Nafion) are commonly used to enhance the adhesion of the electrocatalyst to conductive supports to form electrodes [25–27]. Despite allowing ionic conduction and water management, the polymeric binders always lead to mass and electron transfer limitations, thereby decreasing the overall performance of the electrode [28]. In this work, a sandblasting process was successfully adopted as a binder-free coating approach to enable strong adhesion of electrocatalytic particles on metal substrates (Fig. 3a) [18].

This technique employed corundum particles (median size 110 µm) and used localized energy to transfer catalytic coatings to metallic substrates. After loading the **NiCoV** catalyst at 20:1 weight ratio on the corundum particles (Figure S6), the mixture was blasted onto the surface of the meshes using a pressure of ~ 50 psi N_2_ flow. A dark layer of coating can be visualized by naked eyes after the sandblasting process (Fig. 3b). SEM images of the **NiCoV** coated mesh had a rougher surface with a homogenous coating on the metal surface (Fig. 3c), in which the catalyst can be clearly identified at higher magnifications (Fig. 3d).

**Fig. 4:**
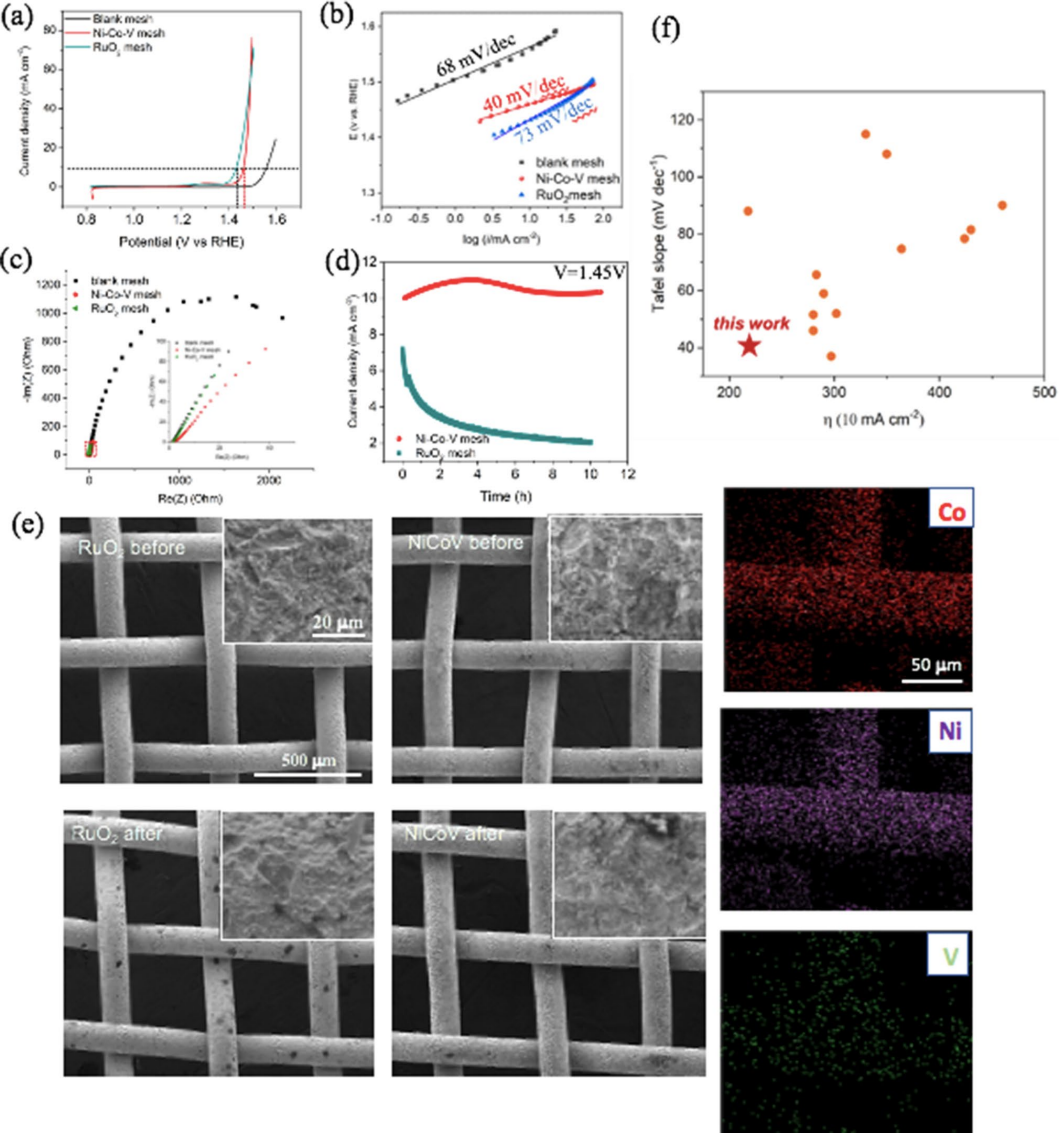
Electrochemical characterization. **a** polarization curves (*iR*-corrected) of catalyst coated stainless steel meshes (sandblasted); **b** Tafel plots for catalyst coated meshes; **c** Nyquist plots of blank mesh, **NiCoV** and RuO_2_ coated mesh. **d** chronoamperometric stability test of RuO_2_ and **NiCoV** coated meshes for 10 h at 1.45 V. **e** SEM images of RuO_2_ (left column) and **NiCoV** coated meshes (right column) before and after the chronoamperometric stability tests, and the EDS mapping was for the sample of NiCoV after stability test. **f** summary of the ŋ (10 mA cm^−2^) and the Tafel slope values of reported Co- and Ni- based OER catalysts from the references, and the red star represents this work (details are available in Table S3). The electrolyte is 1 M KOH

The electrocatalytic performance of the catalyst coated meshes for OER was tested in 1 M KOH. The **NiCoV** coated meshes displayed a decreased overpotential (230 mV), in contrast to the Nafion-**NiCoV** loaded glassy carbon electrode (250 mV) at a current density of 10 mA cm^−2^ (Fig. 4a). This decrease in overpotential may arise from various reasons such as tighter contacts between the **NiCoV** catalyst and stainless steels, which enhances the electron transfer pathway from the catalytically active sites to the highly conductive metal substrate and the absence of superacid Nafion polymer [29]. Although RuO_2_ coated meshes exhibited a relatively lower overpotential (190 mV) compared to the **NiCoV** counterpart, the smaller Tafel plots values suggest the excellent OER performance of the **NiCoV** coated mesh (40 mV dec^−1^) even compared to the commercial RuO_2_ coated mesh (73 mV dec^−1^) (Fig. 4b).In Fig. 4c, electrochemical impedance spectroscopic (EIS) measurements of the samples show that the **NiCoV** coated meshes showed a similar charge transfer resistance (*R*_*ct*_ **NiCoV** ~ 0.345 Ω) compared to the RuO_2_ coated mesh (*R*_*ct*_ RuO_2_ ~ 0.311 Ω), and a lower charge transfer resistance than a blank stainless-steel mesh (*R*_*ct*_ stainless steel ~ 2.169 Ω). Additionally, other control catalysts have been coated on the meshes using the same sandblasting process. We found that the VO^2+^ doping lowered the charge transfer resistance (i.e., *R*_*ct*_ **NiV** ~ 0.231 Ω *c.f. R*_*ct*_ **Ni** ~ 5.433 Ω; *R*_*ct*_ **NiCoV** ~ 0.345 Ω *c.f. R*_*ct*_ **NiCo** ~ 0.873 Ω) (Figures S7, S8), probably caused by the formation of percolation pathway in amorphous materials (i.e., VO^2+^ doped samples: **NiV** and **NiCoV**) that improves the ionic diffusion [21].

Chronoamperometric measurements were carried out in 1 M KOH to test the stability of the catalyst coated meshes at 1.45 V for 10 h. The current density of the **NiCoV** coated mesh showed 7.2% loss, whereas RuO_2_ coated mesh decreased by 71.4% (Fig. 4d). This loss of activity appears to be resulting from a loss of RuO_2_ materials after 10 h chronoamperometric test (Fig. 4e). In contrast, NiCoV coatings remain intact with no material detachment despite a strong gas evolution at the mesh surface during the electrolysis.

Beside morphological preservation, the chemical compositions of post-OER NiCoV coated mesh has been confirmed unchanged by EDS (Fig. 4e). All the results suggested that the tribochemical coatings formed through sandblasting is a method of choice to prepare physically durable coatings.

We finally benchmarked the performance metrics of the **NiCoV** against reported Co-, Ni- based OER electrocatalysts in Fig. 4f and Table S3. Most of the transition metal (Co and Ni) based catalysts have the overpotential range from 250–450 mV (at current density of 10 mA cm^−2^) with the Tafel slope ranging from 50–100 mV dec^−1^. The prepared **NiCoV** coated mesh electrode in this work showed a superior performance (230 mV, 40.0 mV dec^−1^) compared to the majority of the reported catalysts so far. Additionally, the catalyst coated meshes also exhibited high structural and compositional stability after extended periods of oxygen evolution (10 h). Given the low overpotential and Tafel slope and high electrochemical durability, this cheap amorphous catalytic coating is a strong competitor to commercial RuO_2_ and could become a key factor in electrolyzer implementation.

## Conclusion

In summary, we developed a simple low temperature combustion process to synthesize a non-noble ternary electrocatalyst NiCoV (10:80:10, at.%), which exhibited low overpotential 250 mV at a current density of 10 mA cm^−2^ with the Tafel slope of 130 mV dec^−1^ on a glassy carbon electrode. Then binder-free coatings with strong adhesion were fabricated through the sandblasting method, and the NiCoV coated stainless steel mesh displays an extraordinarily low overpotential (230 mV) at a current density of 10 mA cm^−2^ and Tafel slope of 40 mV dec^−1^. No obvious detachment or dissolution of the NiCoV coatings has been detected after 10 h at a potential of 1.45 V, paving the way for promising large-scale oxygen and hydrogen generation.

### Supplementary Information

Below is the link to the electronic supplementary material.

Supplementary file1 (DOCX 7693 KB)

## Data Availability

The data and materials supporting the findings will be made available upon reasonable request to the corresponding author.
