# Supplementary material for: Amorphous multimetal based catalyst for oxygen evolution reaction
Source: Discov Mater. 2024 Jun 25;4(1):19. doi: 10.1007/s43939-024-00087-5 (PMC11199262; doi:10.1007/s43939-024-00087-5)
Supplement: Supplementary file 1 — Supplementary file1 (DOCX 7693 KB) [file 43939_2024_87_MOESM1_ESM.docx]

Amorphous multimetal based catalyst for oxygen evolution reaction

*Zishuai Zhang****^#^****, Daniela Vieira****^#^****, Jake E Barralet****^‡^****, Geraldine Merle****^‡^******

Z. Zhang, D. Vieira, Prof. J. E. Barralet, Prof. G. Merle

Faculty of Medicine, McGill University, Montreal, H3A 0C5, Canada

Current affiliation Prof. G. Merle,

Email: geraldine.merle@polymtl.ca

Chemical Engineering Department, Ecole Polytechnique de Montréal, P.O. Box 6079 Station, Montreal, QC H3C 3A7, Canada

***^#^*** These authors contributed equally to this work

*** Corresponding author

**Table S1.** Compositions for screening NiCoV catalysts and their corresponding potentials at the current density of 10 mA cm^-2^.

| Samples | Ni^2+^ (at. %) | Co^2+^ (at. %) | VO^2+^ (at. %) | η (10 mA cm^-2^) (V vs. RHE) | Tafel slope (mV dec^-1^) |
| --- | --- | --- | --- | --- | --- |
| 1 | 100 | 0 | 0 | 1.53 | 215 |
| 2 | 0 | 100 | 0 | 1.57 | 151 |
| 3 | 40 | 60 | 0 | 1.59 | 290 |
| 4 | 15 | 80 | 5 | 1.52 | 168 |
| 5 | 10 | 85 | 5 | 1.51 | 168 |
| 6 | 80 | 10 | 10 | 1.57 | 214 |
| 7 | 60 | 30 | 10 | 1.57 | 202 |
| 8 | 30 | 60 | 10 | 1.51 | 161 |
| 9 | 10 | 80 | 10 | 1.48 | 130 |
| 10 | 5 | 85 | 10 | 1.49 | 156 |
| 11 | 5 | 80 | 15 | 1.51 | 142 |
| 12 | 10 | 75 | 15 | 1.52 | 157 |
| 13 | 25 | 40 | 35 | 1.52 | 177 |
| 14 | 35 | 30 | 35 | 1.54 | 163 |
| 15 | 30 | 35 | 35 | 1.54 | 172 |
| 16 | 25 | 40 | 35 | 1.53 | 157 |
| 17 | 20 | 45 | 35 | 1.56 | 168 |
| 18 | 10 | 50 | 40 | 1.57 | 152 |
| 19 | 20 | 40 | 40 | 1.56 | 164 |
| 20 | 30 | 30 | 40 | 1.58 | 193 |
| 21 | 40 | 20 | 40 | 1.73 | 260 |
| 22 | 50 | 10 | 40 | 1.74 | 270 |
| 23 | 25 | 35 | 40 | 1.61 | 166 |
| 24 | 15 | 45 | 40 | 1.59 | 167 |
| 25 | 60 | 0 | 40 | 1.76 | 420 |
| 26 | 0 | 60 | 40 | 1.57 | 157 |
| 27 | 50 | 0 | 50 | 1.8 | 450 |
| 28 | 40 | 10 | 50 | 1.74 | 222 |
| 29 | 10 | 40 | 50 | 1.59 | 172 |
| 30 | 20 | 30 | 50 | 1.58 | 173 |
| 31 | 30 | 20 | 50 | 1.69 | 216 |
| 32 | 20 | 10 | 70 | 1.69 | 206 |
| 33 | 10 | 20 | 70 | 1.65 | 187 |
| 34 | 0 | 0 | 100 | >1,70 | >500 |

**Table S2**. Compositions of single metallic catalysts (Ni, Co, V), binary metallic catalysts (NiCo, NiV, CoV) and ternary metallic catalysts (NiCoV).

| Catalyst abbreviation | Ni^2+^ (at. %) | Co^2+^ (at. %) | VO^2+^ (at. %) |
| --- | --- | --- | --- |
| **Ni** | 100 | 0 | 0 |
| **Co** | 0 | 100 | 0 |
| **V** | 0 | 0 | 100 |
| **NiCo** | 40 | 60 | 0 |
| **NiV** | 60 | 0 | 40 |
| **CoV** | 0 | 60 | 40 |
| **NiCoV** | 10 | 80 | 10 |

**Table S3**. Summary of the OER overpotentials (at a current density of 10mA cm^-2^) and Tafel slope of reported Co- and Ni- based OER catalysts.

| Catalyst | Substrate | Solution | Overpotential (mA, at 10mA cm^-2^) | Tafel slope | Ref. |
| --- | --- | --- | --- | --- | --- |
| NiCoV/mesh | Stainless steel mesh | 1M KOH | 220 | 40 | This work |
| RuO_2_/mesh | Stainless steel mesh | 1M KOH | 190 | 73 | This work |
| Ni_3_S_2_/NF | Ni foam | 1M KOH | 350 | 108 | [[1]](https://paperpile.com/c/oV3b0D/AD5H) |
| CoS_2_ nanoparticles | Ni foam | 1M KOH | 430 | 81.4 | [[2]](https://paperpile.com/c/oV3b0D/UO75) |
| CoSe_2_ nanoparticles | Ni foam | 1M KOH | 424 | 78.3 | [[2]](https://paperpile.com/c/oV3b0D/UO75) |
| Co(S_0.22_Se_0.78_)_2_ | Ni foam | 1M KOH | 283 | 65.6 | [[2]](https://paperpile.com/c/oV3b0D/UO75) |
| MoS_2_/ Ni_3_S_2_ | Ni foam | 1M KOH | 218 | 88 | [[3]](https://paperpile.com/c/oV3b0D/ZEiJ) |
| Ni_2_P nanoparticles | Glassy carbon | 1M KOH | 290 | 59 | [[4]](https://paperpile.com/c/oV3b0D/zT3k) |
| FeNiO | Ni foam | 1M KOH | 297 | 37 | [[5]](https://paperpile.com/c/oV3b0D/C8fz) |
| NiCoP/C nanobox | Glassy carbon | 1M KOH | 330 | 115 | [[6]](https://paperpile.com/c/oV3b0D/QTBO) |
| Ni_3_FeN nanosheets | Glassy carbon | 1M KOH | 280 | 46 | [[7]](https://paperpile.com/c/oV3b0D/nHqO) |
| Ni_3_B | Glassy carbon | 1M KOH | 302 | 52 | [[8]](https://paperpile.com/c/oV3b0D/8sbf) |
| NiCo_2_O_4_ nanowires | Conductive electrode | 1M KOH | 460 | 90 | [[9]](https://paperpile.com/c/oV3b0D/cTmI) |
| Co_9−x_Ni_x_S_8_ nanocages | Conductive electrode | 1M NaOH | 364 | 74.7 | [[10]](https://paperpile.com/c/oV3b0D/dr52) |
| P-Co_3_O_4_ | Conductive electrode | 1M KOH | 280 | 51.6 | [[11]](https://paperpile.com/c/oV3b0D/J6Vx) |

**Figure S1**. Electrochemical performance of RuO_2_ catalysts on glassy carbon electrodes. (a) polarization curves of oxygen evolution reaction for different catalysts at a scan rate of 20 mV s^-1^ in 1M KOH, and (b) Tafel slopes of RuO_2_ catalysts on glassy carbon electrodes.


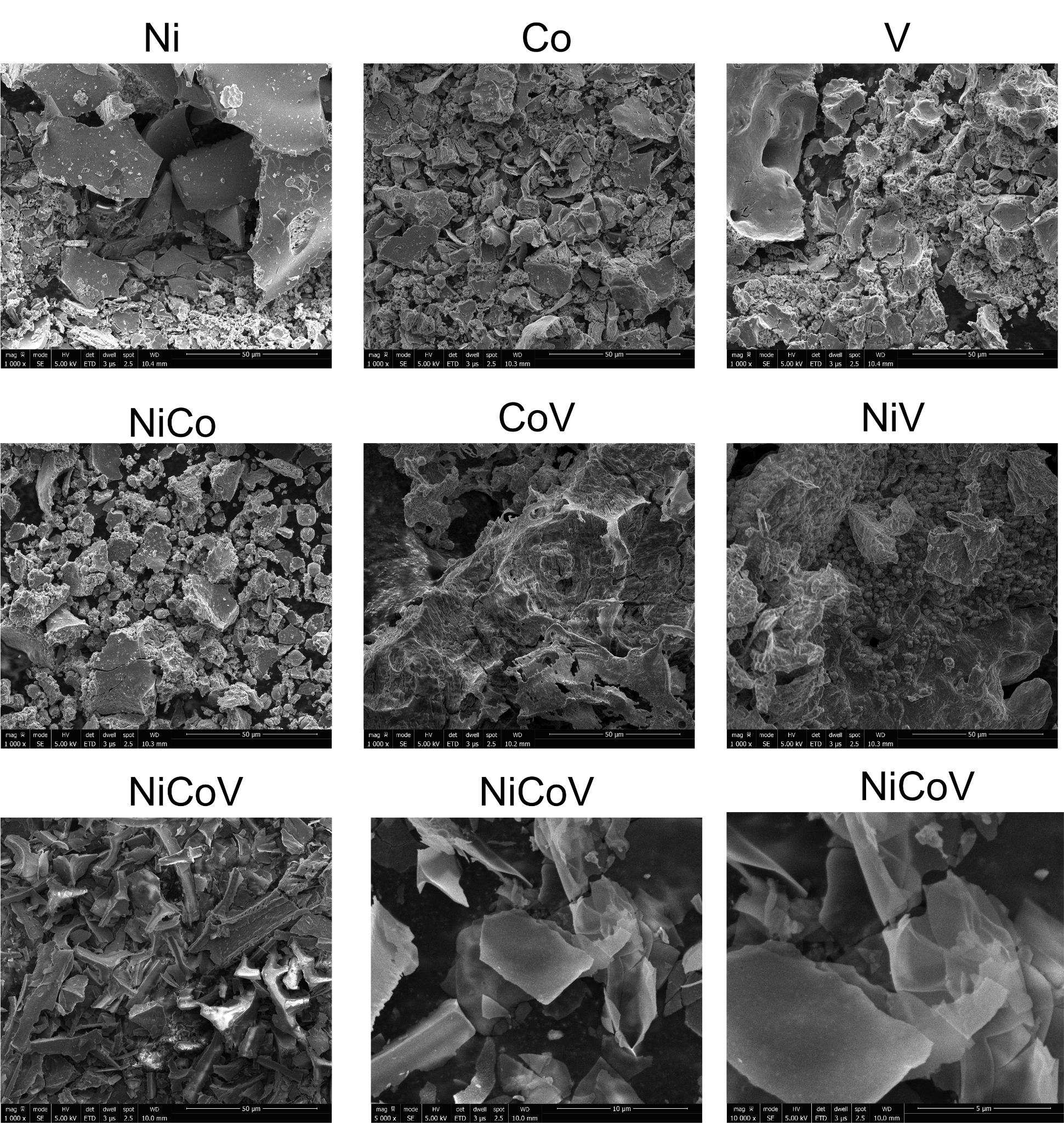


**Figure S2**. SEM images of various components of Ni, Co, V based catalysts. See details about each sample component in Table S2.


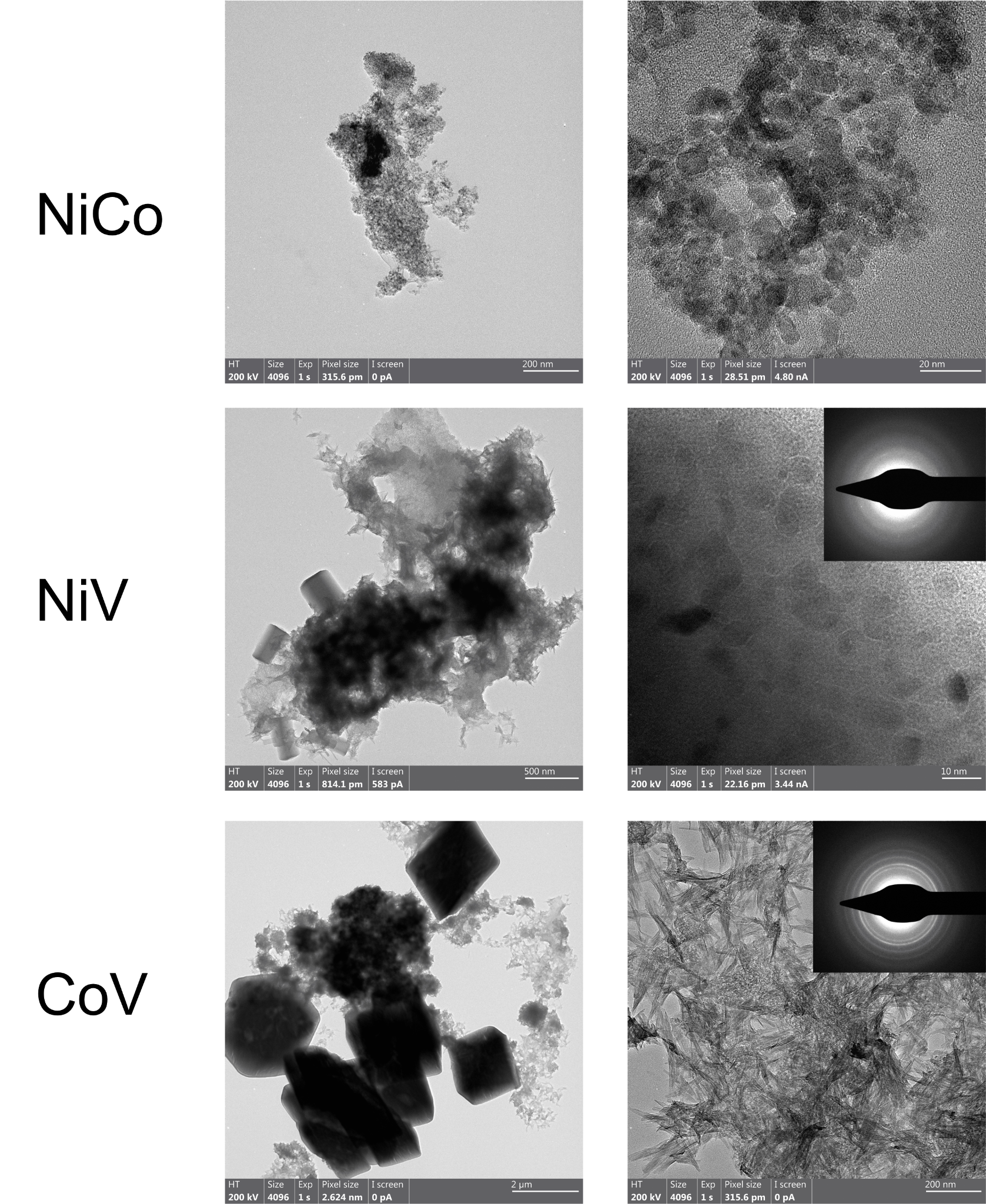


**Figure S3**. TEM images of NiCo, NiV and CoV.


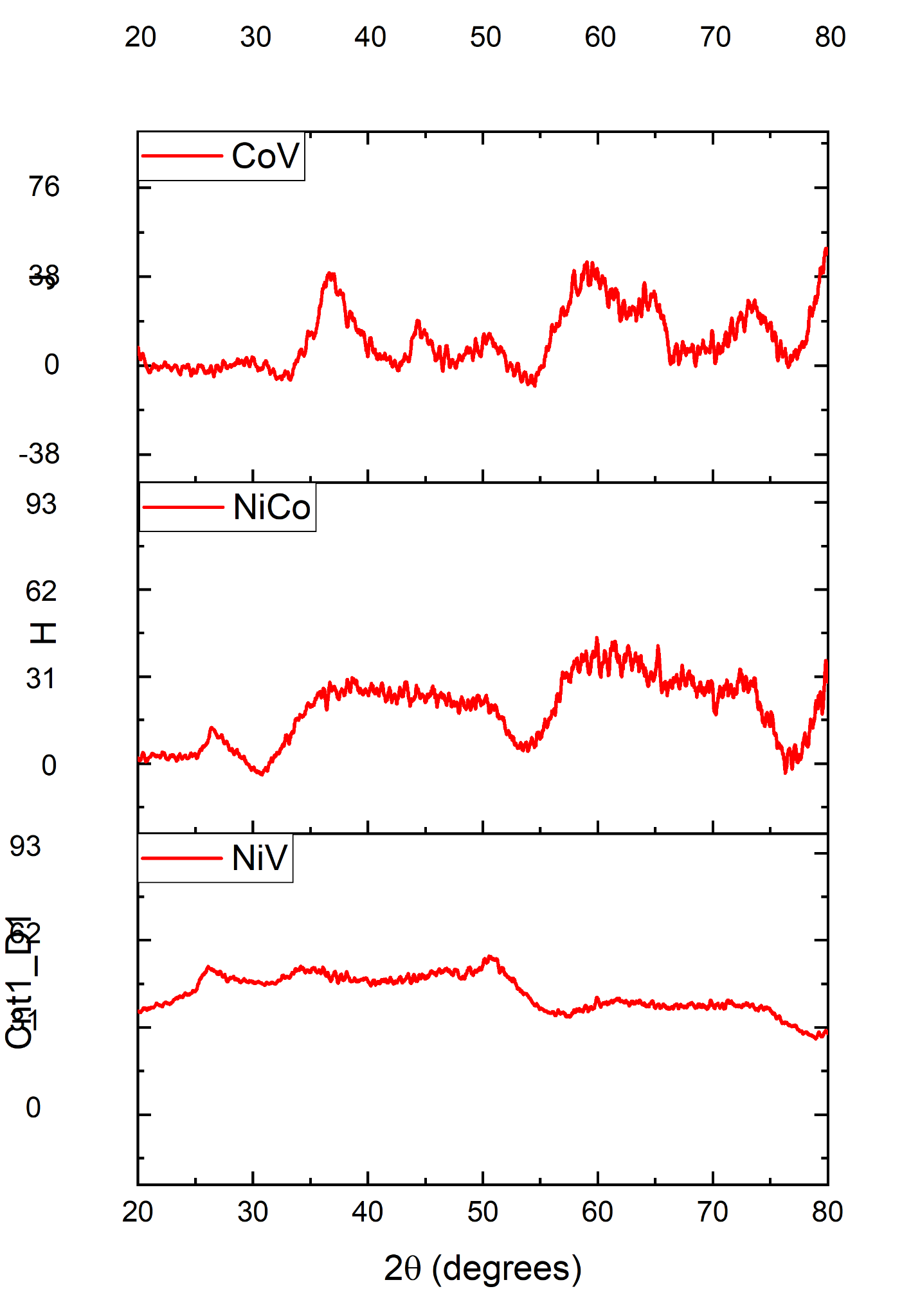


**Figure S4**. XRD patterns of **CoV**, **NiCo** and **NiV** samples. **NiV** shows less pronounced crystalline structure compared to **NiCo** and **CoV**.


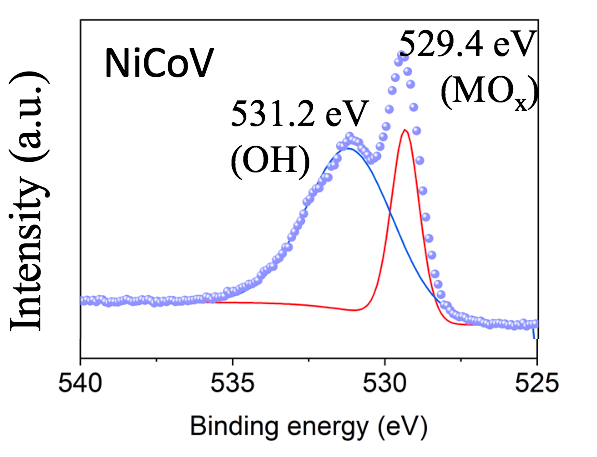


**Figure S5**. High resolution XPS spectra of O 1s for the **NiCoV** sample.


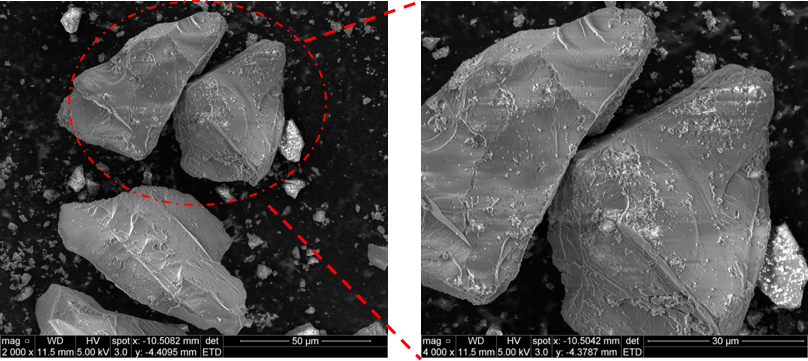


**Figure S6**. SEM images of corundum coated by NiCoV catalyst.

**
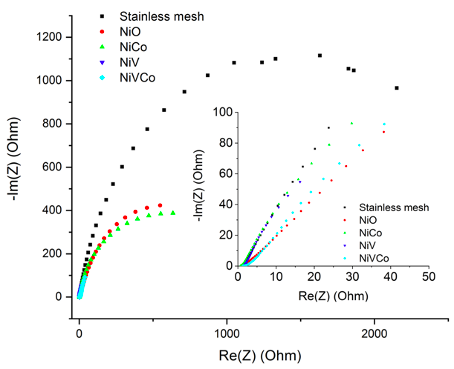
**

**Figure S7.** Nyquist plots of blank mesh, **Ni**, **NiCo**, **NiV** and **NiCoV** coated meshes.


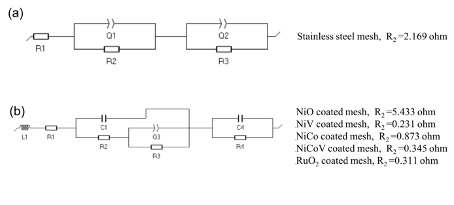


**Figure S8**. Corresponding equivalent electrical circuits for stainless steel mesh (a) and catalyst coated mesh (b) based on the Nyquist plots.

[1] [M. Dincă, Y. Surendranath, D.G. Nocera, Nickel-borate oxygen-evolving catalyst that functions under benign conditions, Proc. Natl. Acad. Sci. U. S. A. 107 (2010) 10337–10341.](http://paperpile.com/b/oV3b0D/AD5H)

[2] [L. Fang, W. Li, Y. Guan, Y. Feng, H. Zhang, S. Wang, Y. Wang, Tuning unique peapod-like co(S x Se1- x )2 nanoparticles for efficient overall water splitting, Adv. Funct. Mater. 27 (2017) 1701008.](http://paperpile.com/b/oV3b0D/UO75)

[3] [J. Zhang, T. Wang, D. Pohl, B. Rellinghaus, R. Dong, S. Liu, X. Zhuang, X. Feng, Interface Engineering of MoS2 /Ni3 S2 Heterostructures for Highly Enhanced Electrochemical Overall-Water-Splitting Activity, Angewandte Chemie International Edition. 55 (2016) 6702–6707. https://doi.org/](http://paperpile.com/b/oV3b0D/ZEiJ)[10.1002/anie.201602237.](http://dx.doi.org/10.1002/anie.201602237.)

[4] [L.-A. Stern, L. Feng, F. Song, X. Hu, Ni2P as a Janus catalyst for water splitting: the oxygen evolution activity of Ni2P nanoparticles, Energy & Environmental Science. 8 (2015) 2347–2351. https://doi.org/](http://paperpile.com/b/oV3b0D/zT3k)[10.1039/c5ee01155h.](http://dx.doi.org/10.1039/c5ee01155h.)

[5] [K. Fominykh, P. Chernev, I. Zaharieva, J. Sicklinger, G. Stefanic, M. Döblinger, A. Müller, A. Pokharel, S. Böcklein, C. Scheu, T. Bein, D. Fattakhova-Rohlfing, Iron-doped nickel oxide nanocrystals as highly efficient electrocatalysts for alkaline water splitting, ACS Nano. 9 (2015) 5180–5188.](http://paperpile.com/b/oV3b0D/C8fz)

[6] [B. Liu, H.-Q. Peng, C.-N. Ho, H. Xue, S. Wu, T.-W. Ng, C.-S. Lee, W. Zhang, Mesoporous Nanosheet Networked Hybrids of Cobalt Oxide and Cobalt Phosphate for Efficient Electrochemical and Photoelectrochemical Oxygen Evolution, Small. 13 (2017). https://doi.org/](http://paperpile.com/b/oV3b0D/QTBO)[10.1002/smll.201701875.](http://dx.doi.org/10.1002/smll.201701875.)

[7] [X. Jia, Y. Zhao, G. Chen, L. Shang, R. Shi, X. Kang, G.I.N. Waterhouse, L.-Z. Wu, C.-H. Tung, T. Zhang, Ni3FeN Nanoparticles Derived from Ultrathin NiFe-Layered Double Hydroxide Nanosheets: An Efficient Overall Water Splitting Electrocatalyst, Adv. Energy Mater. 6 (2016) 1502585.](http://paperpile.com/b/oV3b0D/nHqO)

[8] [W.-J. Jiang, S. Niu, T. Tang, Q.-H. Zhang, X.-Z. Liu, Y. Zhang, Y.-Y. Chen, J.-H. Li, L. Gu, L.-J. Wan, J.-S. Hu, Crystallinity-modulated electrocatalytic activity of a nickel(II) borate thin layer on Ni3 B for efficient water oxidation, Angew. Chem. Int. Ed Engl. 56 (2017) 6572–6577.](http://paperpile.com/b/oV3b0D/8sbf)

[9] [X. Yu, Z. Sun, Z. Yan, B. Xiang, X. Liu, P. Du, Direct growth of porous crystalline NiCo2O4 nanowire arrays on a conductive electrode for high-performance electrocatalytic water oxidation, J. Mater. Chem. A. 2 (2014) 20823–20831. https://doi.org/](http://paperpile.com/b/oV3b0D/cTmI)[10.1039/c4ta05315j.](http://dx.doi.org/10.1039/c4ta05315j.)

[10] [J. Kim, H. Jin, A. Oh, H. Baik, S.H. Joo, K. Lee, Synthesis of compositionally tunable, hollow mixed metal sulphide CoxNiySz octahedral nanocages and their composition-dependent electrocatalytic activities for oxygen evolution reaction, Nanoscale. 9 (2017) 15397–15406.](http://paperpile.com/b/oV3b0D/dr52)

[11] [Z. Xiao, Y. Wang, Y.-C. Huang, Z. Wei, C.-L. Dong, J. Ma, S. Shen, Y. Li, S. Wang, Filling the oxygen vacancies in Co 3 O 4 with phosphorus: an ultra-efficient electrocatalyst for overall water splitting, Energy Environ. Sci. 10 (2017) 2563–2569.](http://paperpile.com/b/oV3b0D/J6Vx)
